# The Changing Epidemiology of Carbapenemase-Producing Enterobacterales

**DOI:** 10.5041/RMMJ.10461

**Published:** 2022-01-27

**Authors:** Khetam Hussein, Yuval Geffen, Orna Eluk, Sigal Warman, Worood Aboalheja, Tamar Alon, Ibrahim Firan, Mical Paul

**Affiliations:** 1 Infectious Diseases Institute, Rambam Health Care Campus, Haifa, Israel; 2 The Ruth & Bruce Rappaport Faculty of Medicine, Technion–Israel Institute of Technology, Haifa, Israel; 3 Microbiology Laboratory, Rambam Health Care Campus, Haifa, Israel

**Keywords:** Carbapenemase-producing Enterobacteriaceae, Enterobacterales, KPC, NDM, OXA-48

## Abstract

**Objective:**

Israeli hospitals were confronted with a major national outbreak of carbapenemase-producing Enterobacterales (CPE) starting in 2006, caused predominantly by monoclonal *Klebsiella pneumoniae* carbapenemase (KPC)-producing *Klebsiella pneumoniae*. Our hospital, Rambam Health Care Campus (RHCC), was one of the medical centers affected by this outbreak. We aimed to investigate the changing epidemiology of CPE at RHCC since 2006.

**Methods:**

This was a retrospective observational cohort study performed in Northern Israel (Haifa) at RHCC, which is a primary tertiary acute care academic hospital. The study included all patients who had acquired CPE at RHCC between January 2005 and December 2020.

**Results:**

The proportion of patients infected with *K. pneumoniae* dropped from 100% of all CPE in the first years to 28% (37/134) in 2020. In 2014, the carbapenemase in 94% of all CPE patients (89/95) was KPC. This decreased to 56% in 2020, while New Delhi metallo-β-lactamase (NDM) and OXA-48 carbapenemases increased from 4% and 2% to 29% (39/134) and 12.7% (17/134) of CPE, respectively.

**Conclusions:**

The CPE epidemic evolved from KPC-producing *K. pneumoniae* to involve different Enterobacterales and carbapenemases. Our results are a microcosm of the current global epidemiology attesting to globalization in bacteriology. The results have implications for infection control and antibiotic treatment of CPE infections.

## INTRODUCTION

Carbapenem-resistant Enterobacterales (CRE) are amongst the major challenges that have been facing healthcare institutions throughout the world in the last two decades.^1^ Carbapenem resistance among CRE is mediated most commonly by broad-spectrum β-lactamases that hydrolyze and inactivate the β-lactam ring of all known β-lactams, including the last-resort carbapenems. These broad-spectrum enzymes are labeled carbapenemases, and the CRE producing these are named carbapenemase-producing Enterobacterales (CPE). The most common currently known carbapenemases include *Klebsiella pneumoniae* carbapenemase (KPC), New Delhi metallo-β-lactamase (NDM), imipenemase (IMI), oxacillinases (OXA)-48, and Verona integron-encoded metallo-β-lactamase (VIM). Israeli hospitals confronted a major national outbreak starting in 2006, caused predominantly by monoclonal KPC-producing *Klebsiella pneumoniae*.^2^ Following a national intervention, the spread of CRE was contained.^2^

Our medical center, Rambam Health Care Campus (RHCC), located in Northern Israel, was among the affected hospitals. The prevalence of CRE at RHCC increased from 2006, reaching a peak of 186.6 new acquisitions per 100,000 hospital-days in 2008.^3^,^4^ As described nationally, this outbreak was caused by KPC-producing *K. pneumoniae*; from January 2006 to April 2007, all 88 carbapenem-resistant *K. pneumoniae* patient isolates carried KPC.^3^

Some of the carbapenemases are chromosomal, but most often, and notably, the KPCs are found on plasmids, hence they are able to move between bacterial species. Indeed, with time, KPC spread to different Enterobacterales, and the introduction of diverse carbapenemases was noted. The aim of this study was to describe the introduction of new carbapenemases at RHCC and the spread of carbapenemases among Enterobacterales.

## METHODS

The study was conducted at RHCC, a 1,000-bed primary and tertiary university hospital in Northern Israel. All patients who acquired CPE at RHCC between January 2005 and December 2020 were included in the study. At RHCC, CPE was considered as acquired if the positive sample was taken ≥72 hours after admission, within 72 hours after any discharge, or within one month after discharge, if the patient was not hospitalized in another healthcare facility. Patients who acquired CRE before admission to RHCC were excluded.

### Microbiology

Rectal swab screening samples were cultured prior to 2019 on PD420 CHROMagar KPC plates; since 2019, samples were cultured on PD517 MSUPERCARBA plates (both from Hy Laboratories Ltd, Rehovot, Israel). The latter medium was used because of its higher sensitivity to non-KPC CPE. Carbapenemase-producing Enterobacterales was defined as Enterobacterales of any type resistant to all tested carbapenems using the Clinical and Laboratory Standards Institute (CLSI) M100S guidelines definition of MIC>1. We extracted DNA from suspected CRE colonies using the Qiamp DNA mini kit (QIAgen, Hilden, Germany) in accordance with the manufacturer’s instructions. Since 2014, β-lactamase (bla) carbapenemase genes, i.e. *bla_KPC_*/*bla_NDM_*/*bla_OXA-48_*/*bla_VIM_*, have been detected routinely using polymerase chain reaction (PCR)-based multiplexed assays specific for these genes.^5^

## RESULTS

**Figure 1:**
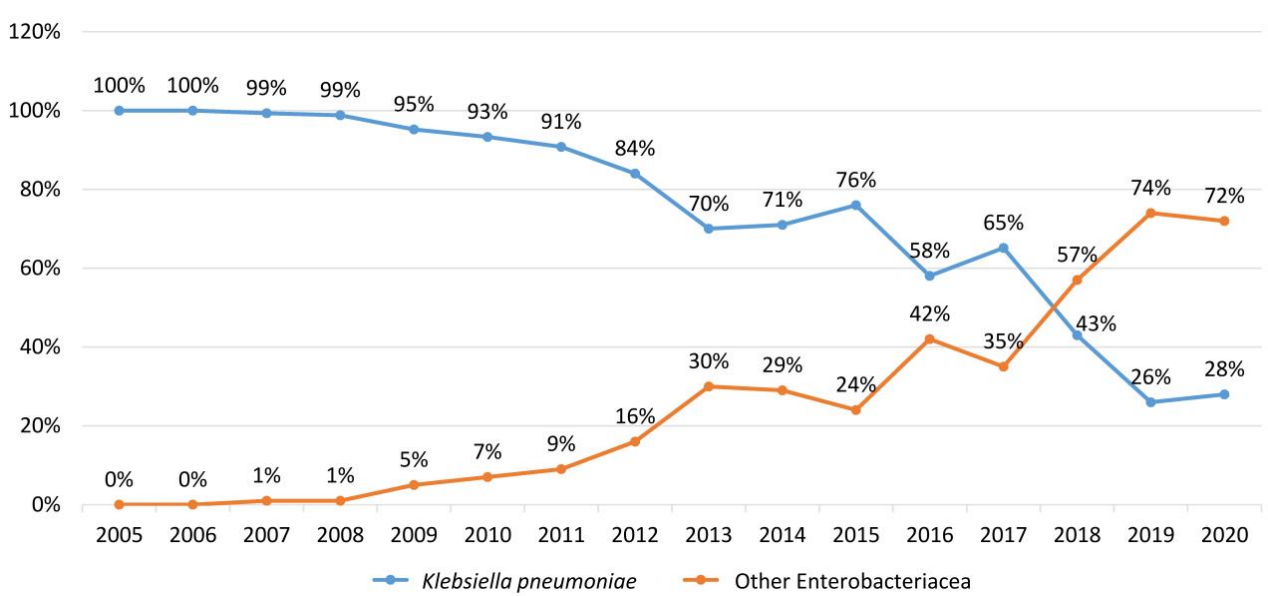
Proportion of CPE Patients diagnosed as carriers of *Klebsiella pneumoniae* versus with Other Enterobacteriacea at Rambam Health Care Campus, Haifa, Israel

Between January 2005 and December 2020, a total of 891,926 patients were admitted to RHCC. During this period, 1,868 patients acquired CPE in RHCC. From 2005 to 2009, almost all isolates were *K. pneumoniae*. Starting in 2009, the proportion of patients carrying CRE with other strains increased gradually, reaching 72% (97/134) in 2020, while the proportion of *K. pneumoniae* decreased to 28% (37/134) in 2020 (Figure 1). The most common Enterobacterales other than *K. pneumoniae* were: *Escherichia coli* and *Enterobacter* spp., followed by *Citrobacter* spp., *K. oxytoca*, *Raoultella* spp., *Morganella* spp., *Proteus* spp., and *Providencia* spp.

The mechanisms of resistance and types of carbapenemases also changed. In 2014, 94% (89/95) of all CPE acquired at RHCC possessed the KPC gene. Only 6% (6/95) of isolates possessed other resistance genes. This proportion decreased to 56% (75/134) in 2020 (Table 1). The proportion of NDM and OXA-48 increased to 29% (39/134) and 12.7% (17/134), respectively (Table 1).

**Table 1:** Distribution of CPE Mechanisms of Resistance Among Newly Diagnosed Patients with CPE Per Type and Year at Rambam Health Care Campus, Haifa, Israel.

Year	Total Number of CPE Patients	KPC *n* (%)	NDM *n* (%)	OXA-48 *n* (%)	IMI *n* (%)
2014	95	89 (94%)	4 (4%)	2 (2%)	0
2015	109	100 (92%)	4 (4%)	5 (5%)	0
2016	59	51 (84%)	3 (5%)	5 (8%)	0
2017	65	58 (88%)	4 (6%)	3 (5%)	0
2018	88	58 (66%)	19 (22%)	11 (13%)	0
2019	141	71 (50.5%)	41 (29%)	27 (19%)	2 (1.5%)
2020	134	75 (56%)	39 (29%)	17 (12.7%)	3 (2.3%)

CPE, carbapenemase-producing Enterobacterales; IMI, imipenemase; KPC, *Klebsiella pneumoniae* carbapenemase; NDM, New Delhi metallo-β-lactamase; OXA, oxacillinases.

## DISCUSSION

We examined the changing epidemiology of CPE in terms of pathogens and resistance mechanisms in one Israeli institution (RHCC) endemic for CPE. Starting from an outbreak confined to KPC-producing *K. pneumoniae*, spread of carbapenemases to other Enterobacterales strains was reported in RHCC as early as 2010.^6^ In 2020, only 28% of all CPE were *K. pneumoniae*, and less than half possessed the KPC gene. Carbapenemases spread to many different Enterobacterales, and new carbapenemases were introduced.

Since the first description of KPC-producing *K. pneumoniae* in North Carolina, USA, in 1996,^7^ KPC-producing *K. pneumoniae* has spread to many locations worldwide.^8^ The KPC gene was subsequently found in other Enterobacteriaceae.^9^ A study using short and long-read sequencing of the carbapenemase-carrying plasmids among Enterobacterales proved plasmid transfer between different Enterobacterales and KPC-containing transposons between plasmids (horizontal transmission), thus explaining the chain of transmission in an outbreak of KPC-producing Enterobacterales in Columbia.^10^ Furthermore, resistance genes have large diversity as described in another study that evaluated the emerging mechanisms of resistance in CRE between 2013 and 2016 at a health system in Northern California, USA. A total of 38.7% of CRE isolates were carbapenemase gene-positive, comprising 25.0% OXA-48, 20.8% KPC, 20.8% NDM, 20.8% SME, 8.3% IMP, and 4.2% VIM.^11^ Although historically the prevalence of carbapenemases had a typical geographic distribution (KPC in USA, Europe, and China; VIM in Greece; OXA-48 in Turkey; and NDM in the Indian subcontinent), these have now spread all over the world.^8^

Our findings are consistent with this spread, which has a major impact on infection control. The diversity of isolates and resistant genes may indicate that, besides clonal transmission of CRE in the hospital, alternative transmission occurs through horizontal spread of mobile genetic elements and plasmids. Modalities to prevent transfer of carbapenemases between Enterobacterales are necessary, possibly avoiding certain antibiotics, such as carbapenems which are direct drivers of carbapenem resistance. While previously all CRE carriers could be cohorted, separate isolation according to the type of carbapenemase is currently necessary. The diverse resistance mechanisms also affect our choice of empirical and definite antibiotic treatment, since the new β-lactamases are carbapenemase-specific.

Our study has some limitations. First, it was conducted in one medical center that was endemic for CPE. Second, we included only CPE acquired in RHCC due to lack of microbiological data and molecular studies on isolates acquired elsewhere. However, 95% of CPE carriers hospitalized in RHCC during the study period had acquired CPE there. This study does not analyze the mechanisms leading to this spread of carbapenemases among bacteria and the reasons leading to the introduction of new carbapenemases to RHCC; only the CPE epidemiology at RHCC over time is described. Similar changes are most likely occurring in other Israeli hospitals. Data published in the 2019 national report for all CPE found in Israeli hospitals reported that only 47% were KPC, 33% were NDM, 12% OXA, 5% VIM, and 3% other; it is likely that these CPE circulate within and among hospitals.^12^

In conclusion, we demonstrate the change in carbapenem resistance mechanisms in one Israeli hospital, the introduction of new carbapenemases, and the spread of carbapenemases across Enterobacterales. These findings, if representative of global epidemiology, are of great concern. Irrespective of location, infection control efforts are essential to prevent the further spread of carbapenemases and CPE.

## Abbreviations

bla: β-lactamase
CPE: carbapenemase-producing Enterobacterales
IMI: imipenemase
KPC: *Klebsiella pneumoniae* carbapenemase
MBL: metallo-β-lactamase
NDM: New Delhi metallo-β-lactamase
IMI: imipenemase
RHCC: Rambam Health Care Campus
VIM: Verona integron-encoded metallo-β-lactamase
